# The effect of complex training and ballistic exercise on the time-course adaptations of lower extremity explosive strength in elite female field hockey players

**DOI:** 10.3389/fpubh.2025.1676079

**Published:** 2025-10-17

**Authors:** Shuo Wang, Xianglong Jiang, Zitong Chen, Xinyang Xing, Xiaofeng Zhang, Tongtong Che

**Affiliations:** ^1^Hebei Sport University, Shijiazhuang, Hebei, China; ^2^School of Physical Education, Qingdao University, Qingdao, China; ^3^Jiangsu Agricultural and Forestry Vocational and Technical College, Zhenjiang, Jiangsu, China

**Keywords:** complex training, ballistic exercise, lower limb explosive power, time-efficiency, female hockey athletes

## Abstract

**Objective:**

To compare the short- and long-term effects of complex training (CT) and ballistic exercise (BE) on lower-limb explosive performance in elite female hockey players and to identify temporal adaptation patterns.

**Methods:**

Twenty-four athletes were randomized to CT (*n* = 8), BE (*n* = 8), or control (CG, *n* = 8). Interventions lasted 8 weeks (3 sessions/week). Performance outcomes included countermovement jump (CMJ), 30-m sprint, and squat 1RM, assessed at baseline, week 4, and week 8.

**Results:**

At week 4, the BE group improved CMJ (*p* < 0.01, *d* = 1.85), sprint (*p* < 0.01, *d* = 0.90), and 1RM (*p* < 0.05, *d* = 0.39), with no further gains by week 8 (*p* > 0.05). The CT group improved sprint (*p* < 0.01, *d* = 0.60) and 1RM (*p* < 0.01, *d* = 0.92) at week 4, while CMJ remained unchanged. By week 8, CT demonstrated significant improvements in CMJ (*p* < 0.05, *d* = 1.24), additional sprint gains (*p* < 0.01, *d* = 0.51), and continued 1RM increases (*p* < 0.01, *d* = 1.72). The CG declined in CMJ (*p* < 0.05, *d* = 0.44) and 1RM (*p* < 0.01, *d* = 1.03), with no sprint changes. Between groups, BE outperformed CG in CMJ at week 4 (*p* < 0.05). At week 8, both CT (*p* < 0.01) and BE (*p* < 0.05) exceeded CG in CMJ; sprint favored CT and BE over CG (*p* < 0.05); and 1RM favored CT over BE (*p* < 0.05) and CG (*p* < 0.01).

**Conclusion:**

BE induces rapid short-term improvements in jumping and sprinting but plateaus after 4 weeks. CT produces slower yet sustained gains across all indicators by 8 weeks, with superior strength development. BE is suited for pre-competition phases, whereas CT is preferable during preparatory periods for integrated strength–power adaptation.

## Introduction

Hockey, a competitive sport conducted under uniform field conditions, requires athletes to integrate physical fitness and technical skills amidst intense competition. Characterized by the execution of high-intensity actions such as sprints, jumps, directional changes, and shots (1), these maneuvers are pivotal in determining athletic performance (2). Lower-limb explosiveness has emerged as a core determinant influencing game outcomes, with a significant positive correlation between vertical jump height and sprint speed among high-level athletes (3). Consequently, hockey places unique demands on the coordinated explosiveness of the hip, knee, and ankle joints. These demands highlight the critical role of developing lower-limb power to meet the high physical intensity of elite-level competition.

Traditional strength training methods, while effective in enhancing basic strength, demonstrate limited transfer to sport-specific explosive actions (4). To address this limitation, researchers have explored targeted methods such as complex training (CT) and ballistic exercise (BE). CT integrates high-load resistance training with rapid stretch-shortening exercises, spanning the force–velocity spectrum to enhance both strength and power (5, 6). Previous studies have shown that training 2–3 times per week for 6–10 weeks (7), and specifically 8 weeks (8), produces significant improvements in sprinting and vertical jump performance. BE, by contrast, involves accelerated movements through the entire concentric range, often with load release at the endpoint (9–11), and typically employs lighter loads to maximize high-velocity force production (12). This approach has been widely applied in sports requiring explosive lower-limb actions (13).

However, research on CT and BE has been limited in several key areas. Most studies have focused on male athletes or single-sport populations, leaving limited evidence available for elite female hockey players. In addition, the way performance adaptations evolve over different training durations remains insufficiently explored. From a theoretical perspective, the principle of training specificity emphasizes that adaptations are maximized when training stimuli closely match the biomechanical and neuromuscular demands of the sport (14). Moreover, mechanistic studies on post-activation performance enhancement (PAPE) indicate that both acute potentiation and longer-term neuromuscular adaptations are time-dependent (15, 16). Together, these frameworks provide a strong rationale for hypothesizing distinct performance responses to CT and BE depending on the duration and nature of the intervention.

To address these gaps, the present study compares the effects of CT and BE on sprinting, jumping, and change-of-direction performance in elite female hockey players across different training durations. This investigation aims to (i) expand the evidence base for explosive training in female hockey, (ii) clarify the temporal patterns of performance adaptation, and (iii) provide practical guidance for optimizing training programs. We hypothesize that: (H1) both CT and BE will improve lower-limb explosive performance; (H2) CT, by integrating force- and velocity-based stimuli, will elicit greater long-term gains; and (H3) BE will induce more rapid early-phase improvements due to its velocity-dominant nature.

## Materials and methods

### Sample size

An *a priori* sample size calculation was performed using G*Power (Version 3.1.9.7; Franz Faul University, Kiel, Germany) based on effect sizes (Cohen’s *d* = 0.8–1.2) reported in previous studies employing repeat sprint and explosive performance protocols with CT and BE interventions (17–19). The analysis indicated that a minimum of 21 participants would provide 80% power to detect significant between-group differences. To account for potential attrition, 24 participants were recruited. All performance testing was conducted during the follicular phase of the menstrual cycle (days 1–10) to minimize potential hormonal confounding effects.

### Participants

Twenty-four elite female hockey players from the Jiangsu Provincial Women’s Field Hockey Team were recruited in collaboration with their coaching staff. All athletes volunteered for the study after receiving a detailed explanation of the objectives, procedures, and potential risks, and all provided written informed consent prior to participation.

Inclusion criteria: (1) age between 18 and 28 years; (2) a minimum of 5 years of structured competitive field hockey experience; (3) proficiency in barbell squat, squat jump, and complex training techniques; (4) relative barbell squat and deadlift strength ≥ 1.5 × body weight; (5) no musculoskeletal injuries in the past 6 months.

Exclusion criteria: (1) any acute or chronic injuries that could affect performance; (2) use of sports supplements, alcohol, caffeine, or other substances that might influence performance or recovery during the intervention; (3) inconsistent participation in training sessions during the study period.

Participants were randomly assigned in equal numbers (*n* = 8 per group) to the Ballistic Exercise (BE) group, Complex Training (CT) group, or Control group (CG) using a computer-generated randomization list prepared by an independent researcher.

All participants continued their standard physical and technical training schedules under the supervision of their team coaches throughout the eight-week intervention. Baseline statistical tests confirmed no significant differences in anthropometric variables, age, or other key indicators among the three groups (*p* > 0.05; see Table 1).

**Table 1:** Baseline characteristics of participants (pre-test results).

Items	CT (*n* = 8)	BE (*n* = 8)	CG (*n* = 8)	*F*	*P*
Height (cm)	167.67 ± 5.79	171.20 ± 5.12	167.33 ± 2.16	1.153	0.344
Weight (kg)	58.62 ± 4.98	60.38 ± 4.23	60.78 ± 3.86	0.407	0.673
Age	20.67 ± 1.86	21.2 ± 2.39	22.67 ± 2.42	1.287	0.307
BMI	20.82 ± 0.75	20.84 ± 0.71	21.7 ± 1.14	1.807	0.200
WHR	0.78 ± 0.02	0.79 ± 0.01	0.79 ± 0.01	0.291	0.752

CT, complex training group; BE, ballistic exercise group; CG, control group; BMI, body mass index; WHR, waist-to-hip ratio.

All procedures were conducted in accordance with the Declaration of Helsinki and were approved by the Academic and Ethics Committee of Hebei Institute of Physical Education (approval number: 2024A13). The participants were informed that all data collected would be processed anonymously.

### Experimental design and exercise protocol

This study adopted a randomized controlled pre-post design with three parallel groups: a Complex Training (CT) group, a Ballistic Exercise (BE) group, and a Control group (CG). The intervention lasted 8 weeks, with all training sessions conducted three times per week under the supervision of a certified strength and conditioning specialist.

The training protocols for each group are summarized in Table 2. Each training session lasted approximately 60 min and included a standardized warm-up (5 min of jogging, mobility drills, and progressive activation exercises). The following sections provide additional details about the experimental and control conditions to ensure replicability.

**Table 2:** Training intervention programs for the CT, BE, and CG groups.

Groups	Training movements	Training loads	Number of reps	Number of sets	Inter-set intervals	Training frequency
CT	Squat	85%1RM	5	5	5 min	3 times/week
	SSC	Bodyweight	5	5	5 min	3 times/week
BE	Weighted Squat Jumps	30%1RM	8	5	5 min	3 times/week

CT, complex training group; BE, ballistic exercise group; CG, control group; SSC, stretch-shortening cycle; RM, repetition maximum; reps, repetitions.

The CT group performed high-load back squats paired with bodyweight plyometric drills in the same session to maximize post-activation potentiation. Specifically, each set of back squats consisted of 5 repetitions performed at 85% of 1RM, followed by a 3-min rest, and then 5 repetitions of plyometric exercises. The plyometric component included countermovement jumps, drop jumps from a 30-cm box, and bounding exercises. A 5-min inter-set rest was provided between sets. The CT protocol emphasized maximal explosive power through combined strength and plyometric movements.

The BE group focused on maximal velocity during weighted squat jumps at 30% of 1RM, with an emphasis on maximal acceleration through the concentric phase of the squat. The set protocol included 8 repetitions per set, with 5 sets and a 5-min rest between sets. The BE protocol was designed to maximize explosive power and develop fast-twitch muscle fibers using submaximal loads.

The CG group continued their normal in-season training program, which included resistance exercises such as barbell squats, bench press, pull-downs, and core stabilization exercises performed at 60–70% of 1RM for 2–3 sets of 8–12 repetitions. Their routine also included aerobic conditioning (20–30 min of running or interval training at moderate intensity) and dynamic flexibility and mobility drills. The CG group maintained their regular in-season routine without the incorporation of additional explosive power training. No additional high-intensity explosive exercises (such as plyometrics or complex training) were incorporated in the CG program.

Training loads were adjusted weekly for both the CT and BE groups based on updated 1RM estimations and RPE (Rate of Perceived Exertion) feedback. The RPE was recorded after each session to assess the intensity of the exercises. In addition, resting morning heart rate (HR) was monitored daily, and session RPE (sRPE) was recorded approximately 30 min after each training session to reflect the athletes’ perceived exertion. When elevated HR or high sRPE values were observed compared with baseline, minor adjustments were made (such as reducing the number of sets or extending recovery intervals), but the core training intensity, exercise type, and frequency remained unchanged.

To minimize variability in the study, all participants were instructed to maintain consistent diet, hydration, and sleep schedules throughout the intervention. They were also instructed to avoid using supplements, alcohol, caffeine, or any other ergogenic aids during the study period. Furthermore, all testing sessions were conducted at the same time of day, by the same staff, using the same equipment and standardized protocols to ensure consistency across all groups. These procedures ensured that potential external factors did not affect the experimental outcomes, contributing to the replicability of the study.

### Testing method

Key tests related to lower limb athletic performance were selected through literature analysis and expert consultations. The tests included the following:

#### Maximum lower limb strength test

Back squat strength was assessed using a standardized 3RM protocol on a free-weight squat rack (Eleiko, Halmstad, Sweden). Participants squatted to approximately 90° knee flexion under supervision. After a general warm-up, participants performed sets at ~50%, ~70%, and ~80% of estimated 1RM (7–10, 5–7, and 3–5 repetitions, respectively; 2 min inter-set rest), followed by 3–4 trials of 3RM with 4 min rest between attempts. The heaviest successful 3RM was used to predict 1RM using the Epley formula. This submaximal prediction protocol has demonstrated excellent test–retest reliability (ICC = 0.97–0.99; CV = 2.2–10.1%). and strong validity compared with direct 1RM testing in trained athletes (20). Additionally, the internal consistency of this method, as well as its predictive validity for 1RM strength, has been well-documented in the literature. Studies have consistently shown that submaximal 3RM testing methods like this have strong correlation with direct 1RM testing, validating their application in similar studies.

#### Explosive strength test

Lower-limb explosive power was measured using a force platform (Kistler Quattro Jump, Type 9,290 AD; Kistler, Winterthur, Switzerland). Participants performed CMJs with hands on hips, starting from an upright stance, executing a countermovement to at least ~90° knee flexion before a maximal vertical jump. A standardized warm-up was completed, followed by three familiarization jumps with ~2 min rest. During the test, participants completed three CMJ trials with 2 min rest, and the best jump height was used for analysis. This protocol has shown excellent reliability (ICC = 0.934; CV = 15.3–22.2%) in athletic populations (21). Moreover, the internal consistency of the CMJ test in measuring lower-limb explosive power has been supported by research indicating strong correlation between trials, ensuring the reliability of this measure across repeated tests.

#### Meter sprint test

Sprint performance was evaluated over 30 m using electronic timing gates (Brower Timing Systems, Draper, UT, United States) set at 0 m and 30 m at ~1.0 m height to detect torso passage. Participants used a standing start 0.3 m behind the first gate to avoid early triggering. After a standardized warm-up (light jogging and dynamic stretching), each participant performed two sprint trials separated by 2–3 min rest, and the fastest time was recorded. This system has demonstrated high test–retest reliability (ICC = 0.96–0.99; CV = 0.7–1.9%) in sprint performance monitoring (22). The internal consistency of the system for sprint timing has been well-documented, showing minimal variability across repeated trials, further supporting its accuracy and reliability in performance testing.

### Statistical analysis

All data were analyzed using SPSS version 26.0. The Shapiro–Wilk test was used to verify data normality. One-way repeated-measures ANOVA was applied to examine within-group changes over time for CMJ height, 30-meter sprint time, and back squat 1RM. One-way ANOVA was used to assess between-group differences at each time point. When significant main effects were detected, Bonferroni-adjusted post-hoc tests were conducted for pairwise comparisons.

The significance of within-group Δ values was tested using paired-sample *t*-tests. This study reports the effect sizes (ES) and their 95% confidence intervals (95% CI) for all primary outcomes to assess the precision and reliability of the magnitude of effects. Between-group comparisons used *η*^2^ (eta-squared) and its CI, *η*^2^ was thresholds interpreted as follows: <0.06 (small), <0.14 (moderate), and ≥0.14 (large) (23). While within-group comparisons used Cohen’s *d* and its CI, Cohen’s *d* was interpreted as follows: <0.2 as trivial, 0.2–0.6 as small, 0.6–1.2 as moderate, 1.2–2.0 as large, and >2.0 as very large (24). Statistical significance was defined as *p* < 0.05 or *p* < 0.01.

## Results and discussion

### Results

#### Pre-test comparison of performance indicators

As shown in Table 3, the baseline athletic performance data for the three groups (CT, BE, and CG) prior to the experimental intervention. A one-way ANOVA revealed no statistically significant differences between the groups in countermovement jump (CMJ) height (*F* = 0.483, *p* = 0.627), 30-meter sprint time (*F* = 0.563, *p* = 0.582), or 1-repetition maximum (1RM) squat strength (*F* = 0.011, *p* = 0.989). The associated effect sizes (*η*^2^) were small, and their 95% confidence intervals all included zero, further confirming the lack of meaningful baseline differences. This establishes that the groups were equivalent in terms of lower-limb explosive strength and maximal strength before the training period, ensuring that any subsequent changes in these measures can be attributed to the differential effects of the training interventions.

**Table 3:** Pre-test comparison of performance indicators between groups.

Items	CT	BE	CG	*F*	*P*	*η* ^2^	95% CI
CMJ/cm	41.17 ± 5.23	39.4 ± 4.72	38.33 ± 5.09	0.483	0.627	0.044	(0.000,0.256)
30 m/s	4.85 ± 0.41	4.65 ± 0.07	4.77 ± 0.29	0.563	0.582	0.051	(0.000,0.271)
Squat 1 RM/kg	110.33 ± 9.97	111.00 ± 9.57	110.33 ± 5.85	0.011	0.989	0.003	(0.000,0.152)

CT, complex training group; BE, ballistic exercise group; CG, control group; SSC, stretch-shortening cycle; RM, repetition maximum;95% CI, 95% confidence intervals.

#### Within-group comparison of test indicators

##### Countermovement jump (CMJ) height

**Figure 1:**
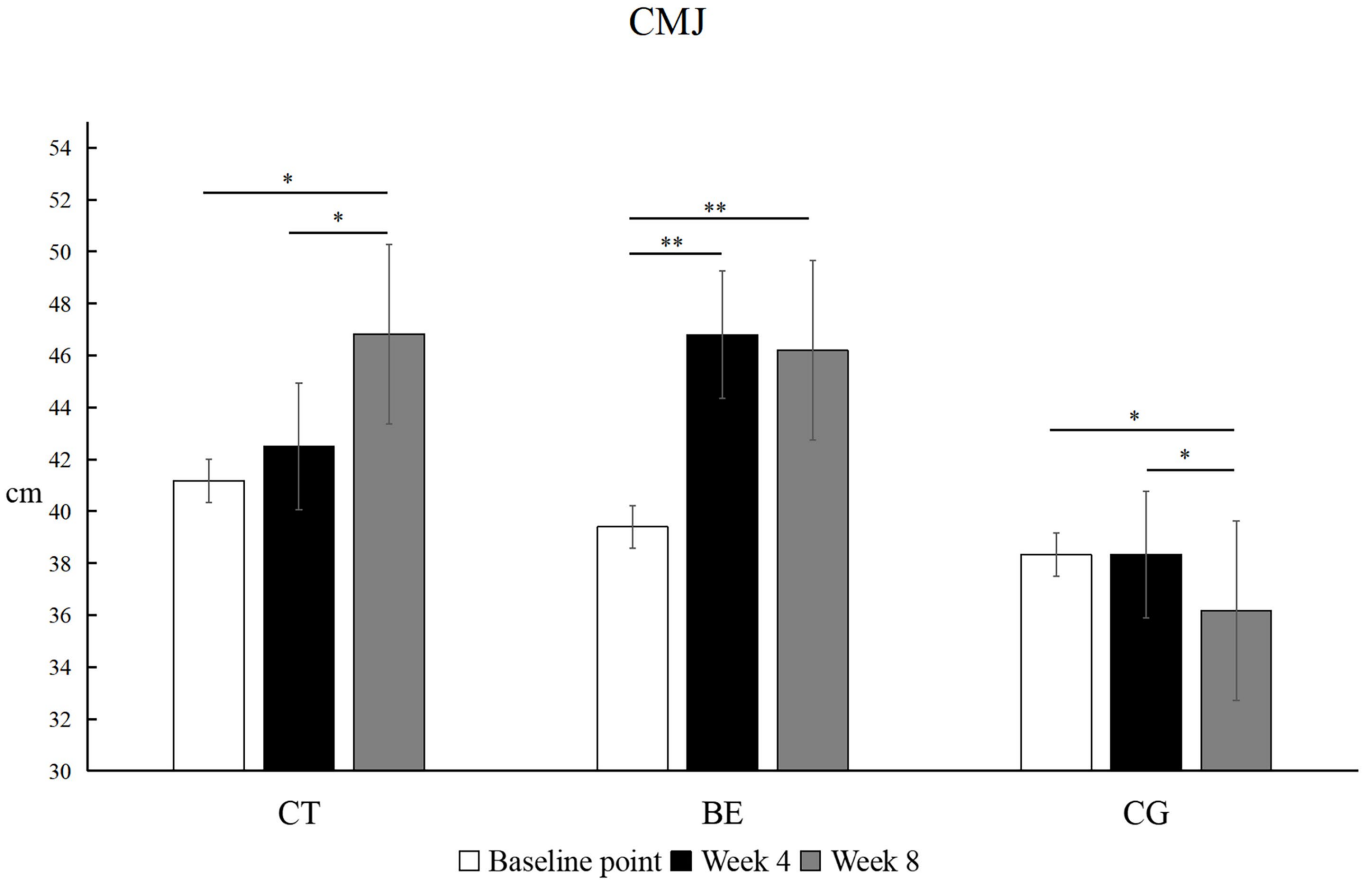
Within-group comparison of CMJ performance. *p<0.05, **p<0.01; unmarked items indicate no significant correlation.

As shown in Figure 1, the within-group comparison of CMJ height revealed distinct temporal adaptations. The Ballistic Exercise (BE) group showed a highly significant improvement from baseline after 4 weeks (*p* < 0.01, *Δ* = +7.40 cm, *d* = 1.85, 95% CI [0.98, 2.72]), but no further change was observed between weeks 4 and 8 (*p* > 0.05, *d* = 0.15, 95% CI [−0.78, 0.48]), indicating a plateau. The Complex Training (CT) group displayed a delayed but progressive response: the change at week 4 was not statistically significant (*p* > 0.05, *Δ* = +1.33 cm, *d* = 0.28, 95% CI [−0.15, 0.71]), but significant improvements were observed by week 8 compared with both baseline and week 4 (*p* < 0.05, Δ = +5.66 cm, *d* = 1.24, 95% CI [0.09, 2.37]). The Control group (CG) showed a significant decrease in CMJ height at week 8 compared with baseline (*p* < 0.05, *Δ* = −2.16 cm, *d* = 0.44, 95% CI [−0.13, 1.01]).

##### 30-meter sprint test

**Figure 2:**
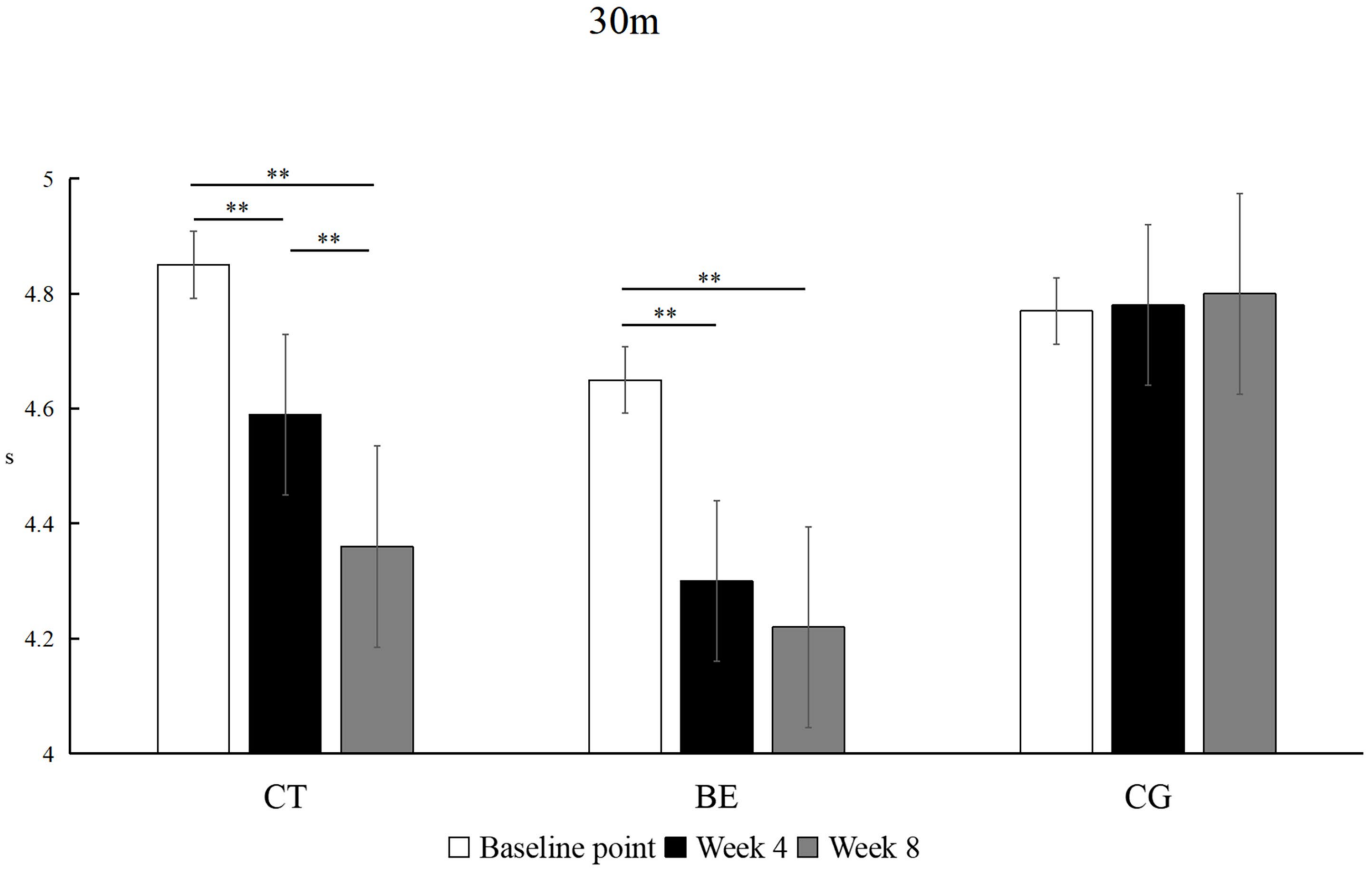
Within-group comparison of 30-m sprint performance. **p<0.01; unmarked items indicate no significant correlation.

As shown in Figure 2, sprint times improved significantly in both intervention groups but followed different trajectories. The CT group demonstrated continuous improvement, with significant reductions from baseline to week 4 (*p* < 0.01, *Δ* = −0.26 s, *d* = 0.60, 95% CI [0.16, 1.03]) and from week 4 to week 8 (*p* < 0.01, *Δ* = −0.23 s, *d* = 0.51, 95% CI [0.08, 0.94]). The BE group showed a rapid initial improvement, with a large effect at week 4 (*p* < 0.01, *Δ* = −0.35 s, *d* = 0.90, 95% CI [0.27, 1.52]), but no further gains by week 8 (*p* > 0.05), although performance remained significantly better than baseline (*p* < 0.01, *Δ* = −0.43 s, *d* = 0.80, 95% CI [0.18, 1.41]). The CG did not exhibit any significant changes across the testing periods (*p* > 0.05).

##### Maximum strength test

**Figure 3:**
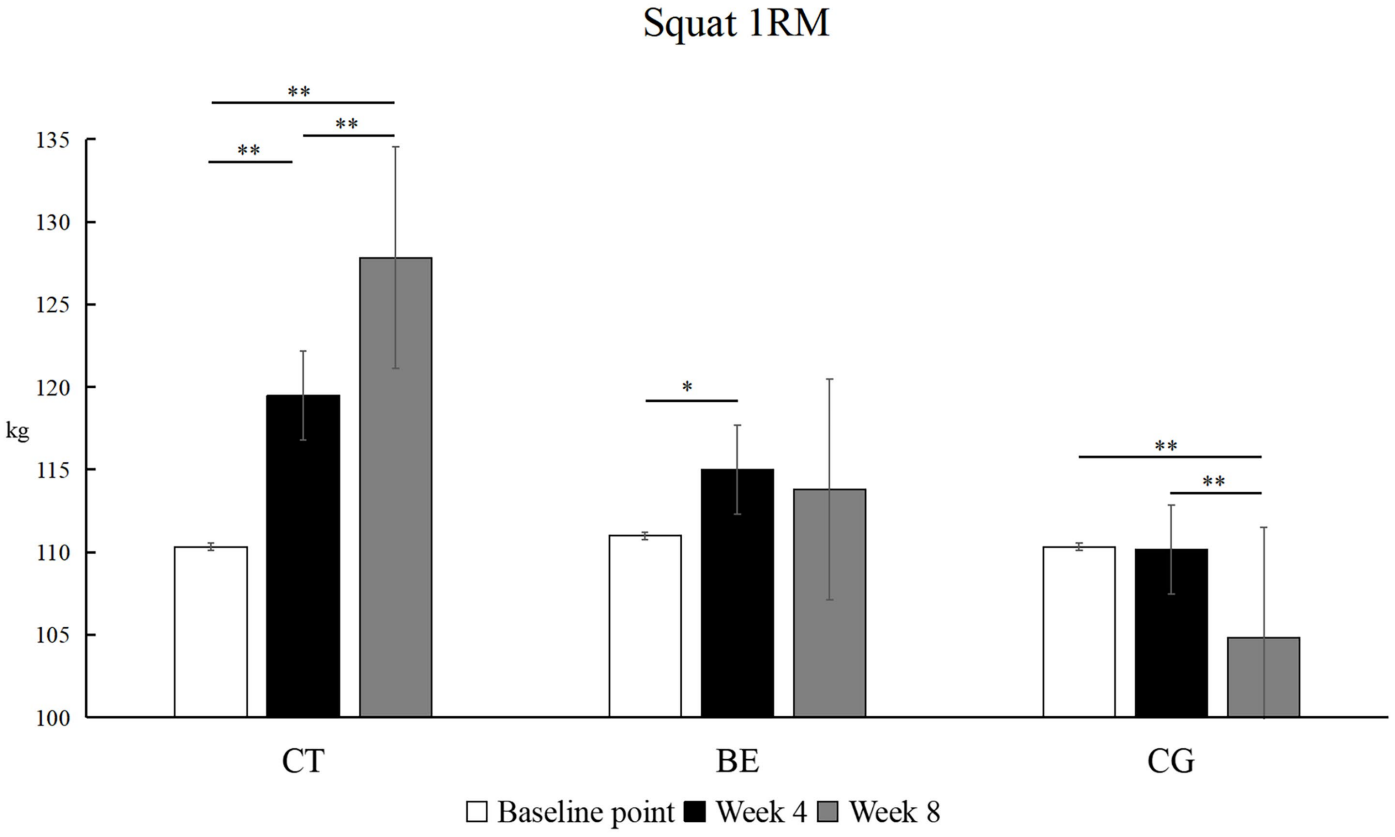
Within-group comparison of back squat 1RM. *p<0.05, **p<0.01; unmarked items indicate no significant correlation.

As shown in Figure 3, lower-limb maximum strength exhibited divergent patterns across groups. The CT group showed consistent linear growth, with significant improvements at every stage (Baseline to Week 4: *p* < 0.01, *Δ* = +9.17 kg, *d* = 0.92, 95% CI [0.25, 1.58]; Week 4 to Week 8: *p* < 0.01, *Δ* = +8.33 kg, *d* = 0.82, 95% CI [0.16, 1.47]; Baseline to Week 8: *p* < 0.01, Δ = +17.50 kg, *d* = 1.72, 95% CI [0.77, 2.64]). The BE group improved only at week 4 (*p* < 0.05, Δ = +4.00 kg, *d* = 0.39, 95% CI [−0.27, 1.04]), after which performance stabilized (*p* > 0.05). In contrast, the CG showed a significant decrease by week 8 compared with both baseline and week 4 (*p* < 0.01, Δ = -5.50 kg, *d* = 1.03, 95% CI [0.36, 1.69]).

#### Between-group comparison

##### Countermovement jump (CMJ) height

**Figure 4:**
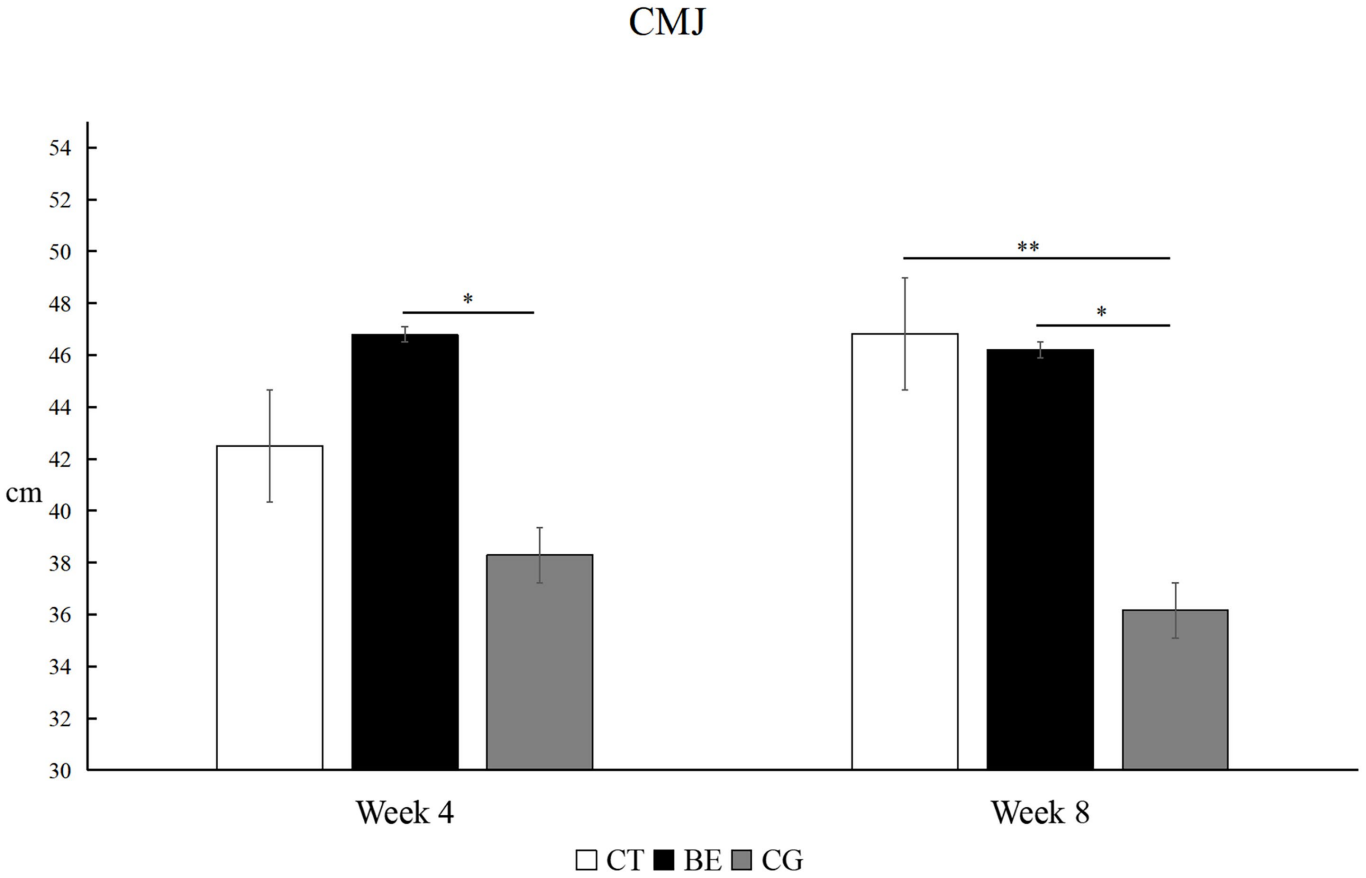
Between-group comparison of CMJ performance. *p<0.05, **p<0.01; unmarked items indicate no significant correlation.

As shown in Figure 4, the between-group comparison of CMJ height revealed a significant difference after 4 weeks (*F* = 6.024, *p* < 0.05), with a large effect size (*η*^2^ = 0.365, 95% CI [0.000, 0.589]). *Post hoc* analysis indicated that the BE group achieved significantly greater CMJ height compared with the CG (*p* < 0.05). After 8 weeks, the between-group difference became highly significant (*F* = 10.933, *p* < 0.01), with a large effect size and narrower confidence intervals (*η*^2^ = 0.451, 95% CI [0.108, 0.642]). *Post ho*c tests further revealed that both the BE and CT groups exhibited significantly greater CMJ height than the CG (BE vs. CG: *p* < 0.05; CT vs. CG: *p* < 0.01), while no significant difference was found between the two training groups (*p* > 0.05).

##### 30-meter sprint

**Figure 5:**
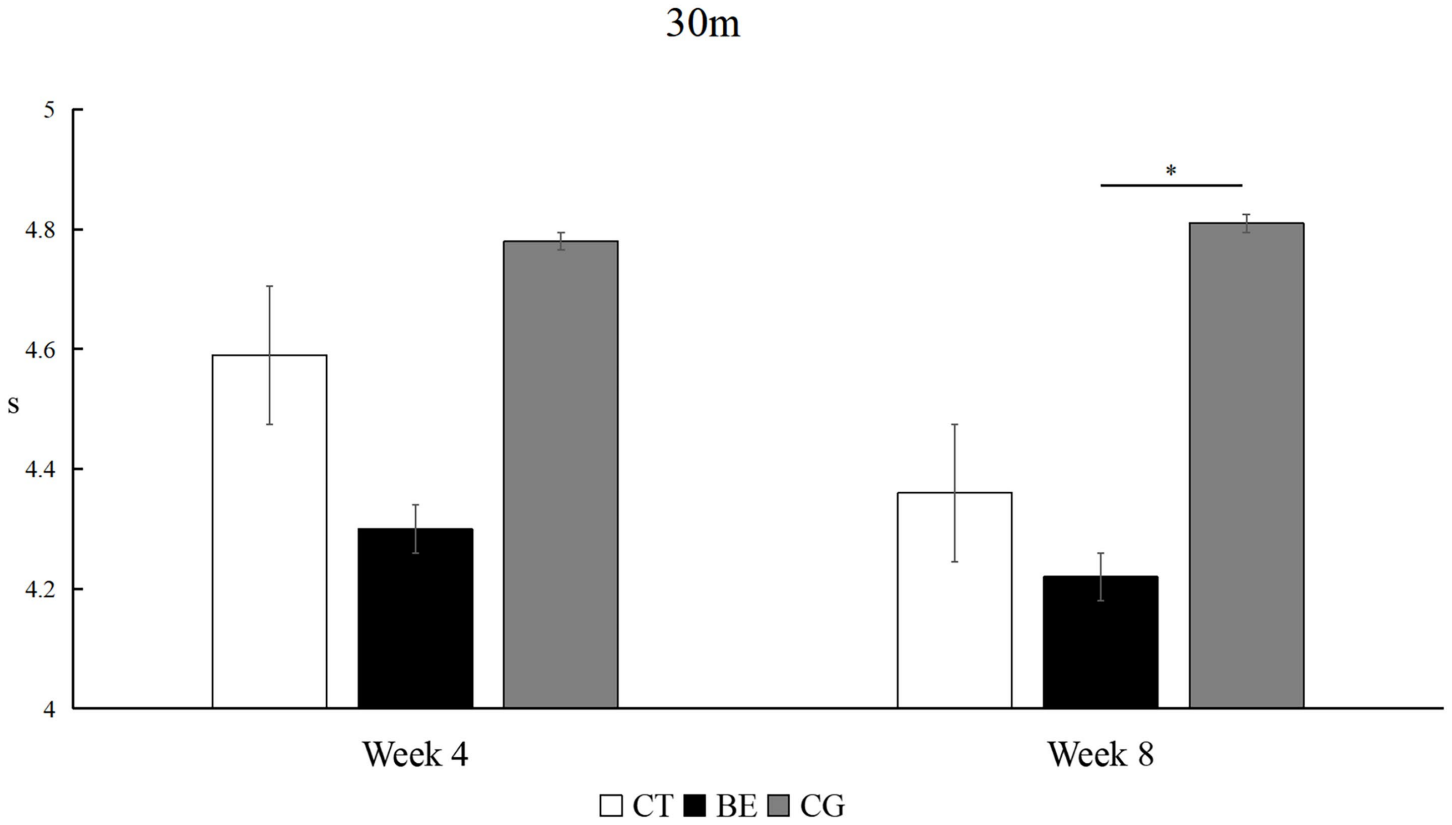
Between-group comparison of 30-m sprint performance. *p<0.05; unmarked items indicate no significant correlation.

As shown in Figure 5, between-group comparisons for the 30-meter sprint showed that after 4 weeks, the difference did not reach overall statistical significance (*F* = 3.047, *p* > 0.05), with a medium but imprecise effect size (*η*^2^ = 0.225, 95% CI [0.000, 0.463]). However, post-hoc analysis revealed that the BE group performed significantly faster than the CG (*p* < 0.05). After 8 weeks, the between-group difference reached statistical significance (*F* = 4.308, *p* < 0.05), with a large effect size (*η*^2^ = 0.291, 95% CI [0.000, 0.531]). *Post hoc* tests indicated that both the BE and CT groups were significantly faster than the CG (*p* < 0.05), while no significant difference was observed between the two training groups (*p* > 0.05).

##### Maximum strength

**Figure 6:**
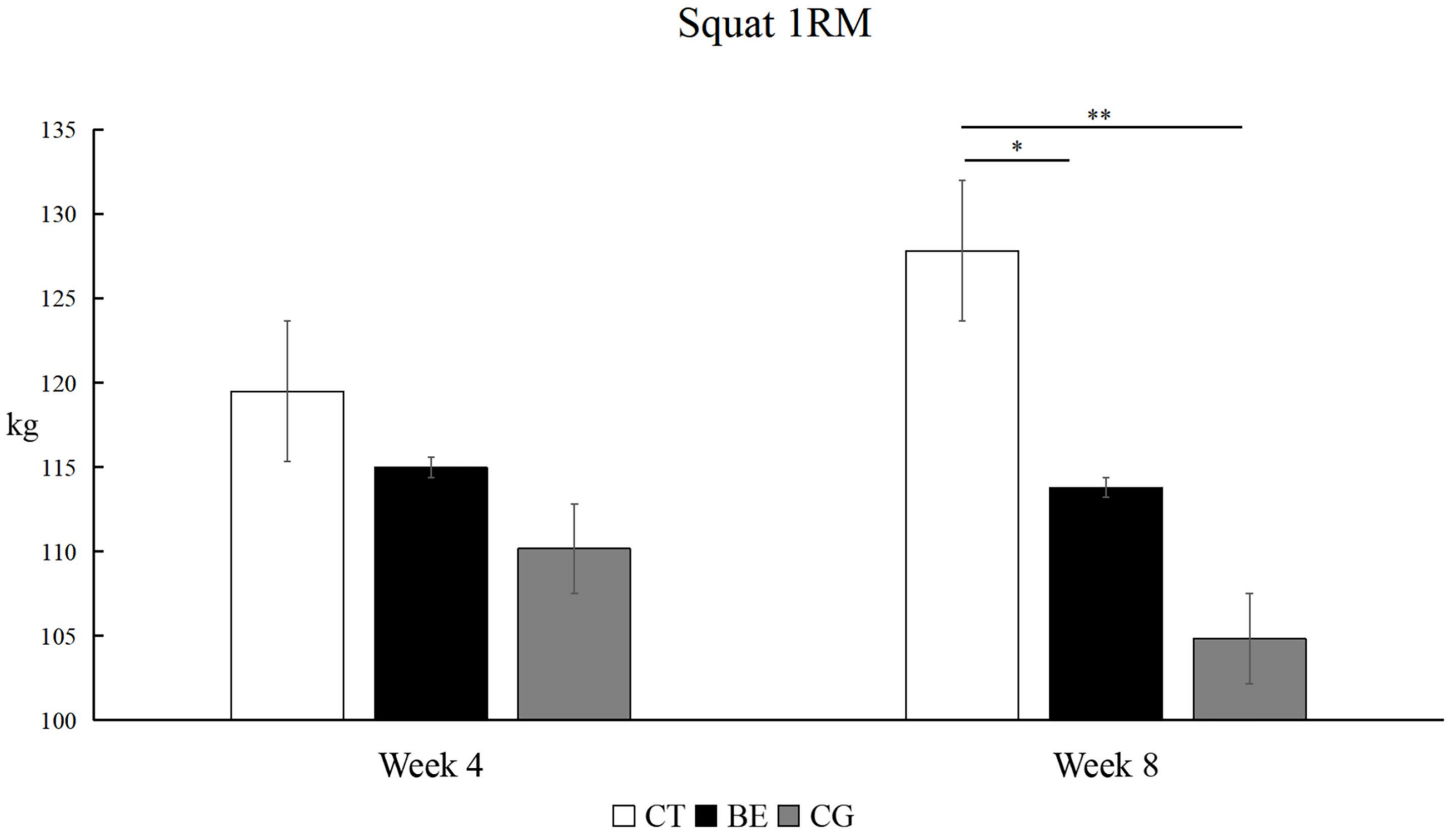
Between-group comparison of back squat 1RM.

As shown in Figure 6, between-group comparisons of back squat 1RM showed no significant difference after 4 weeks (*F* = 1.517, *p* > 0.05), with a small effect size (*η*^2^ = 0.126, 95% CI [0.000, 0.387]). However, after 8 weeks, the between-group difference became highly significant (*F* = 8.611, *p* < 0.01), with a large effect size (*η*^2^ = 0.451, 95% CI [0.108, 0.642]). *Post hoc* tests demonstrated that the CT group had a significantly greater 1RM than both the BE group (*p* < 0.05) and the CG (*p* < 0.01). No significant difference was observed between the BE and CG groups (*p* > 0.05).

## Discussion

### Analysis and discussion of changes in CMJ before and after exercise

Explosive power is fundamentally rooted in absolute strength, with the critical challenge being the effective conversion of absolute strength into functional explosive power (25). The countermovement jump (CMJ) serves as a widely used and pivotal indicator of explosive power, demonstrated to have high reliability and validity (26). In hockey, an athlete’s explosive power directly influences their competitive performance and efficiency, emerging as one of the key determinants of match outcomes. During gameplay, athletes with superior vertical explosive power can more effectively control aerial balls, execute high-position shots, and enhance offensive and defensive initiative. When striking, force is transmitted through a dynamic chain comprising the lower limbs, core, and upper limbs, with the explosive force generated by the lower limbs directly affecting shot speed and strike power.

The most pivotal finding of this study lies in revealing the distinct temporal patterns by which complex training and ballistic exercise influence lower-body explosive power (as measured by the CMJ) in elite female field hockey players. Ballistic exercise exhibits a “rapid onset with early plateauing” pattern, whereas complex training demonstrates a “delayed transition with sustained progression” model. This phenomenon is not coincidental; it stems from the fundamentally different physiological adaptation mechanisms underlying these two training modalities.

First, the remarkable efficacy of 4 weeks of ballistic exercise primarily stems from its highly efficient drive of the nervous system. The findings of this study align closely with those of Haff and Nimphius (27), demonstrating that ballistic exercise (such as 30% of 1RM squat jumps) rapidly optimizes motor unit recruitment efficiency (particularly for high-threshold Type II muscle fibers), increases neuromuscular firing frequency, and enhances intramuscular coordination through its “terminal release” and high-velocity movement characteristics. These neural adaptations occur without significant increases in muscle cross-sectional area, enabling rapid translation into substantial improvements in CMJ height (*d* = 1.85) during the early training phase (within 4 weeks). However, as Maffiuletti et al. (28) point out, this neural adaptation quickly reaches a plateau, due to the lack of sufficient high-intensity resistance loads (e.g., >80% 1RM) to provide sustained stimulation to muscle structure and tendon stiffness, the ceiling for strength reserves is low, leading to diminished subsequent gains (*d* = 0.15 at 8 weeks). This precisely explains why ballistic exercise serves as an effective short-term sharpening tool rather than a long-term foundational program.

Although complex training has been shown to increase vertical jump height (29), this study found no improvement in standing vertical jump height among elite female hockey players after 4 weeks of complex training. The delayed effect of complex training not only embodies the classic theory that “strength is the foundation of explosive power” but also highlights the time cost required for strength to convert into explosive power (30). During the initial phase of complex training (4 weeks), although athletes’ lower-body maximum strength showed significant improvement (*d* = 0.92), the CMJ height only exhibited a small effect increase (*d* = 0.28). This phenomenon does not indicate training ineffectiveness but stems from two factors: First, although 1RM squat correlates with CMJ height, the neuromuscular fatigue induced during the initial phase of high-intensity resistance training temporarily masks actual gains in explosive power. Second, and more critically, the conversion of maximum strength into explosive power (as measured by CMJ performance) depends more on the rate of force production and neuromuscular coordination (4).

Over time, however, technical adaptation and fatigue management allowed significant performance transfer by week 8. This aligns with previous findings from scholars studying long jump athletes (31), the strength gains achieved through complex training require a sufficient duration to be converted into explosive power via specialized training techniques, such as optimizing jump mechanics (13). Suchomel et al. (32) also noted that moderate gains in relative squat strength often require at least 4 weeks of structured training to produce measurable improvements in CMJ performance. This stepwise growth pattern perfectly validates the “Strength–Explosive Power Synergy Cycle” theory and underscores the critical importance of training cycles lasting 8 weeks or longer for power-based events that rely on strength development.

Furthermore, the specificity of sport-specific techniques represents a significant confounding factor in regulating training effects. Our findings diverge from those of Mihalik et al. (33) in volleyball athletes, who achieved a significant increase in vertical jump height after just 4 weeks of complex training. The core reason for this discrepancy lies in sport-specificity: volleyball players frequently perform jumping spikes and blocks in daily training, whose technical patterns closely align with the CMJ. Consequently, they rapidly transfer newly acquired maximal strength to vertical jump performance (i.e., more pronounced training transfer effects). In contrast, hockey players’ specialized techniques primarily involve ground shots and directional acceleration, resulting in relatively lower transfer efficiency of strength to CMJ and requiring longer adaptation periods. This finding further underscores that evaluating training intervention outcomes must account for athletes’ sport-specific contexts to yield scientifically sound conclusions.

### Analysis and discussion of changes in sprint acceleration before and after exercise

Global positioning system tracking analyses of elite hockey matches have consistently demonstrated that 30-meter sprints occur with high frequency during gameplay (34). Short-distance acceleration and explosive power are critical for disrupting defensive formations and creating scoring opportunities. The 30-meter sprint test, which evaluates the ability to reach maximal speed rapidly from a standing start, effectively replicates the three key phases of hockey performance—initial start, acceleration, and early speed maintenance—making it a valid and sport-specific indicator of explosive capacity (35). The performance differences observed in the 30-meter sprint between the two training models in this study can be systematically explained by the force-velocity curve theory (36). This theory reveals that muscle force is inversely proportional to contraction velocity. Training must specifically target different segments of the curve to achieve an overall rightward shift, thereby enhancing speed output under specific loads or force performance at specific speeds. Our findings showed that complex training produced a medium effect size after 4 weeks (*d* = 0.60), with further improvements observed between weeks 4 and 8 (*d* = 0.51), and a large cumulative effect by week 8 (*d* = 1.14). Complex training first builds a maximum strength foundation on the left side of the curve through 85% 1RM squats, followed by SSC to convert strength into speed. This aligns closely with the two-stage model reported by Cormie et al. (4), which involves “broadening strength reserves → optimizing neuromuscular regulation.” This progressive enhancement suggests that complex training requires a longer training duration to fully translate basic strength gains into sport-specific acceleration performance, which is consistent with Villarreal et al. (37), who noted that approximately 8 weeks of progressive strength training are needed for most effective strength-to-power conversion.

In contrast, ballistic exercise (using 30% 1RM squat jumps) prioritizes activating the velocity-strength domain on the right side of the activation curve through high-speed eccentric-concentric contractions. Its substantial effect size at 4 weeks (*d* = 0.90) stems from rapid optimization of neuromuscular drive efficiency in type II muscle fibers. This pattern is consistent with findings from Cormie et al. (4), who emphasized that sustained sprint performance improvements require both neural and muscular adaptations. Female athletes, who typically have smaller muscle cross-sectional areas and lower fast-twitch fiber proportions (5–10% less than males), tend to rely more on neuromuscular coordination than absolute strength for acceleration (38). This explains the BE group’s early improvements in sprint performance but due to the insufficient stimulation of the muscle-tendon complex structure under light loads, its effects plateaued after 8 weeks. This aligns with research findings that while plyometric training can enhance muscle stiffness, it struggles to sustainably increase tendon elastic energy storage capacity (39). The control group which showed no significant changes (*p* > 0.05), may have maintained baseline acceleration ability due to frequent acceleration drills in hockey-specific training. However, without the additional stimulus provided by structured complex training or ballistic exercise, cannot achieve a qualitative breakthrough.

### Analysis and discussion of changes in squat maximal strength before and after exercise

Maximal squat strength is a fundamental indicator of lower limb force production capacity and has long been recognized as a cornerstone of athletic performance (40). Improvements in maximal strength are mediated by physiological and biochemical adaptations of muscle tissue, enhanced neuromuscular coordination, and hormone regulation (41). One critical mechanism is the reduction of neuromuscular inhibition through repeated exposure to high-intensity loading, which increases motor unit recruitment efficiency and reduces antagonist co-activation.

The results of the 1RM squat most directly reveal the fundamental difference between compound and ballistic training: the former relies on both neuromuscular adaptation and morphological adaptation, while the latter primarily depends on neuromuscular adaptation. The complex training group demonstrated large effect size gains at both 4 and 8 weeks (*d* = 0.92, 0.82), clearly demonstrating the advantages of its compound stimulation approach. This is consistent with Roig et al. (42), who emphasized that high-load resistance training induces superior neural drive and strength adaptations compared with low-load protocols. The combination of 85%1RM squats and stretch–shortening cycle exercises in complex training likely leveraged post-activation potentiation (PAP), thereby simultaneously stimulating maximal strength and explosive force output.

In contrast, the ballistic exercise group demonstrated only small improvements (*d* = 0.38), this is entirely as expected. Although the 30% 1RM squat jump activates type II muscle fibers, it lacks sufficient mechanical tension to stimulate muscle cross-sectional area growth, making it difficult to sustainably increase strength reserves. As Suchomel et al. (13) noted, low-load (30% 1RM) ballistic exercise improves motor control but provides insufficient stimulus to increase muscle cross-sectional area, thereby limiting maximal strength gains over extended periods. This divergence between complex training and ballistic exercise suggests that while both methods enhance performance, only complex training provides the necessary overload for sustained strength progression.

One of the most valuable findings of this study lies in the significant decline observed in the control group (*d* = 1.03), which provides an extremely important warning for the physical training of female field hockey players. The significant decline in maximal strength observed may reflect the influence of concurrent aerobic training, particularly during the summer preparation phase when the team’s program emphasized high-volume endurance conditioning to build aerobic capacity. As detailed in the Methods, the control group engaged in aerobic running and interval conditioning three times per week (20–30 min at moderate intensity), combined with resistance training at approximately 60–70% 1RM. Previous studies have shown that endurance-dominant training can activate the AMPK signaling pathway, which interferes with mTOR-mediated protein synthesis and accelerates the selective atrophy of fast-twitch muscle fibers (43). Similarly, Fyfe et al. (44) reported that concurrent endurance training attenuated strength gains, supporting our interpretation in this elite female hockey cohort. Within the specific context of this study, the predominance of aerobic sessions during the summer phase likely amplified this molecular interference, thereby contributing to the observed reduction in maximal strength in the control group. From a practical perspective, these results suggest that incorporating complex training into hockey conditioning programs can counteract the catabolic effects of high-volume aerobic work by maintaining activation of the mTOR pathway and promoting hypertrophic and neural adaptations. In contrast, ballistic exercise alone, while beneficial for neuromuscular coordination, appears insufficient to mitigate the negative impact of concurrent endurance training on maximal strength.

The theoretical significance of this study lies in its first-ever demonstration among female field hockey players that complex training achieves a sustained rightward shift in the strength-velocity curve through the sequential alignment of “structural adaptation-functional transfer.” Meanwhile, the neural adaptation advantage of ballistic exercise requires integration with periodization to yield long-term effects. This provides experimental evidence for resolving the conflict between “short-term explosive power gains and long-term strength reserves,” offering particularly valuable guidance for designing training cycles for female athletes.

### Limitations

This study has certain limitations. First, the relatively small sample size, constrained by the number of available players, may limit the generalizability of the results. A larger sample would enhance the robustness of the results and reduce the potential influence of outliers. Second, the participants were all elite female hockey players, therefore, the observed responses to complex training and ballistic exercise may not be directly transferable to male athletes, recreational populations, or athletes from other sports that impose different neuromuscular demands. Finally, the intervention period was limited to 8 weeks; longer-term studies are needed to examine whether the observed adaptations can be sustained or further enhanced. Despite these limitations, the present findings provide meaningful evidence for the application of complex training and ballistic exercise in elite female hockey players.

## Conclusion

After 4 weeks of intervention, the BE demonstrated significant improvements in the CMJ and 30-meter sprint acceleration, accompanied by a small increase in lower limb maximal strength, though without a statistically significant advantage. The CT exhibited notable enhancements in both 30-meter acceleration and lower limb maximal strength. After 8 weeks of training, the CMJ and 30-meter sprint acceleration of catapult training tended to be stable, and there was no further significant difference compared with 4 weeks. In contrast, the CT continued to demonstrate progress in CMJ and 30-meter acceleration (with no significant difference from the BE group), while their lower limb maximal strength was significantly higher than that of the BE.

### Recommendations

Based on the findings of this study, the following evidence-based recommendations are proposed to support the design of training programs for female hockey athletes:

#### Implement ballistic exercise for rapid power development

Given the acute improvements in explosive performance observed with ballistic training, it is recommended for use during pre-competition phases or short-term training cycles (e.g., 4 weeks). This method enhances neuromuscular coordination and sprint acceleration—qualities essential for hockey performance—and aligns with the time-sensitive demands of competitive preparation.

#### Adopt complex training for long-term athletic development

The sustained improvements in both strength and power support the use of complex training over longer periods (≥8 weeks), particularly during preparatory phases. This approach supports dual development of maximal strength and power through synergistic exercise pairing, making it ideal for structural training periods where cumulative adaptation is prioritized.

#### Maintain consistent resistance training to preserve strength qualities

The observed declines in maximal strength and vertical jump performance following periods of resistance training cessation highlight the need to avoid interruptions longer than 4 weeks—especially during aerobic-dominated training phases. Incorporating regular high-intensity resistance sessions mitigates the negative impact of endurance work and helps maintain critical neuromuscular adaptations.

These recommendations are derived directly from the experimental outcomes and are intended to help coaches and sport scientists structure training in ways that are both time-efficient and physiologically specific to female hockey players.

## Data Availability

The original contributions presented in the study are included in the article/supplementary material, further inquiries can be directed to the corresponding author.
